# SSRI co‐medication with NOAC or VKA does not increase hospitalisation for bleeding: A retrospective nationwide cohort study in Austria 2010‐2015

**DOI:** 10.1002/gps.5117

**Published:** 2019-04-24

**Authors:** Safoura Sheikh Rezaei, Martina Mittlböck, Bertholdt Reichardt, Michael Wolzt

**Affiliations:** ^1^ Department of Clinical Pharmacology Medical University of Vienna Vienna Austria; ^2^ Center for Medical Statistics, Informatics and Intelligent Systems Medical University of Vienna Vienna Austria; ^3^ Sickness Fund Burgenland Eisenstadt Austria

**Keywords:** bleeding events, NOAC, pharmacoepidemiology, SSRI, VKA

## Abstract

**Objectives:**

Non‐vitamin K oral anticoagulants (NOACs) or vitamin K antagonists (VKAs) are used for the prophylaxis and treatment of thromboembolic events. A potential drug–drug interaction and increased bleeding events have been reported with co‐medication of selective serotonin receptor inhibitors (SSRIs) and VKA. The aim of this study was to investigate the bleeding risk of a coprescription of NOAC or VKA with SSRI.

**Methods:**

Patients with prescription of NOAC or VKA and an antidepressant drug therapy (ADTx) were selected from the drug reimbursement database of 13 Austrian health insurance funds. For this cohort, hospital discharge diagnoses for gastrointestinal bleeding, cerebral haemorrhage, and bleeding anaemia between 2010 and 2015 were analysed.

**Results:**

Data were available from 50 196 female and 31 308 male patients. Among these, 892 patients had 987 hospitalisations with bleeding events. The most frequent bleeding cases were gastrointestinal bleedings with 588 events (59.6%), followed by cerebral haemorrhage with 344 (34.8%), and bleeding anaemia with 55 events (5.6%), respectively. The risk of bleeding events was similar between SSRI and other ADTx, when combined with oral anticoagulants (*p* = 0.51). Concomitant treatment of patients with SSRI or other ADTx and NOAC was associated with an increased bleeding risk compared with cotreatment with VKA (1.21, 95% CI: 1.05‐1.40; *p* = 0.0097).

**Conclusion:**

Co‐medication of SSRI with VKA or NOAC has little if any impact on hospital discharge diagnoses for bleeding events compared with cotreatment of those anticoagulants with other antidepressant medications.

**Key points**

Coprescription of SSRI with oral anticoagulants has a similar risk for major bleedings as the combination with other antidepressants.

**Abbreviations**
ADTx: Antidepressant drug therapy
ATC: Anatomical Therapeutic Chemical Classification System
CI: Confidence interval
CV disease: Cardiovascular disease
GI bleeding: Gastrointestinal bleeding
NOACs: Non‐vitamin K oral anticoagulants
RR: Risk ratio
SSRI: Selective serotonin reuptake inhibitor
VKA: Vitamin K antagonist

## 1 INTRODUCTION

The increase in non‐vitamin K oral anticoagulant (NOAC) prescription has raised concern in clinical practice about safety and possible drug–drug interactions with other medication. NOAC and vitamin K antagonists (VKA) are indicated for the prevention of stroke and systemic embolism in atrial fibrillation and for treatment of venous thromboembolism. A strict laboratory monitoring is needed to ensure therapeutic efficacy of VKA. Over the past few years, apixaban, rivaroxaban, dabigatran, and edoxaban have been introduced as NOAC and offer several advantages over VKA.^1^, ^2^ These NOAC mitigate clot formation by interfering selectively with thrombin or factor Xa and have a rapid onset of therapeutic action, shorter half‐life, and predictable pharmacodynamic effects, and do not require routine laboratory monitoring compared with VKA. However, drug–drug interactions need to be considered to avoid an increased bleeding risk.^3^ There is evidence that SSRI may increase bleeding risks, particularly when combined with VKA.^4^ It is unknown if a similar risk is conferred by coadministration of NOAC with SSRI or other antidepressant medication.

Selective serotonin reuptake inhibitors (SSRIs) are widely used in the treatment of depression, especially among older people.^5^, ^6^ In addition to being present on nerve cells, serotonin receptors are also expressed on the surface of platelets, and by inhibiting the reuptake of the agonist, SSRIs prolong the action of serotonin once released. Thus, SSRIs reduce the ability of platelets to aggregate and increase the risk of bleeding.^7^

The aim of the present retrospective epidemiological study was to investigate bleeding events in patients with NOAC or VKA treatment in combination with antidepressant drug therapy (ADTx) in a real world setting. The group of patients receiving SSRI was compared with patients receiving other ADTx.

## 2 MATERIALS AND METHODS

This study was approved by the Ethics Committee of the Medical University of Vienna (EK‐No. 2198/2016) and was performed in accordance with the Declaration of Helsinki.

### 2.1 Data preparation

The health insurance system in Austria provides health care for all residents who are assigned membership to one of the several health insurance funds according to their current or former employment or province of residence. Data from medical services covered by the health insurance funds are stored in the respective databases. These include demographic data, information on hospital discharges with primary diagnoses coded using the ICD‐system and reimbursed drug prescriptions. Each medication is described by the unique Austrian pharmaceutical registration number and linked to the Anatomical Therapeutic Chemical (ATC) Classification System. We analysed data between 2010 and 2015 from Austrian health insurance funds covering about 99% of the Austrian population. Data were pseudonymised to preserve patients' privacy. Data storage and handling were in agreement with data protection laws.

### 2.2 Cohort selection

Patients aged 18 years or older who filled a prescription of NOAC or VKA were eligible for this study. ATC codes and subcodes B01AF01, B01AF02, and B01AE07 identified patients with NOAC and B01AA including subcodes those receiving VKA, respectively. Among these patients, those who reimbursed a prescription of SSRI with ATC codes and subcodes N06AB02, N06AB03, N06AB04, N06AB05, N06AB06, N06AB07, N06AB08, N06AB09, and N06AB10 were selected. We used ATC codes and subcodes N06AA, N06AC, N06AF, N06AG, and N06AX to identify patients with other ADTx. Patients with a maximum of one switch between prescriptions of NOAC and VKA or SSRI and other ADTx were included. Analysed were patients who had a filled prescription with anticoagulant and antidepressant medicine from day one, irrespectively of treatment duration. For simplification, we excluded the small number of patients for whom records indicated simultaneous prescription of different anticoagulant and antidepressant drugs during the observational period. The duration of drug intake was defined as the time between the first and last recorded prescription plus the period of last medication coverage.

### 2.3 Clinical endpoints

Gastrointestinal bleeding (GI bleeding, ie, I85.0, K25‐28, K62.5, K62.6, and K92), cerebral haemorrhage (ie, I61), and bleeding anaemia (D62) were defined as bleeding events by using ICD codes and subcodes from hospital discharge diagnosis during the observation period. Co‐morbidities were defined by ATC codes and subcodes. We used medication classified as A10AB‐A10AF, except for A10B for diabetes, C02, C03, C07, C08, and C09 for cardiovascular (CV) diseases, and R03 for pulmonary diseases (PDs), respectively. An overlapping intake of anticoagulant and antidepressant medication and 30 days thereafter was investigated with respect to an association with bleeding events.

### 2.4 Statistical methods

The continuous variable age was described by median, quartiles and maximum. Categorical data are described by absolute frequencies and percentages. The risk for bleeding events was calculated as the ratio of events and the sum of person years under risk for the predefined treatment courses. To determine the number of events, a generalised linear model with Poisson distribution and log link was used, and the logarithm of person years was used as offset. The possibility of more than one treatment course per patient was modelled with an independent variance–covariance matrix for repeated measurements per patient. Risk differences between groups are described by risk ratios (RRs) and corresponding 95% confidence intervals (95% CIs). All *p* values ≤ 0.05 were considered statistically significant. Statistical analysis was performed by the statistical software SAS (SAS Institute Inc., Cary, NC, USA).

## 3 RESULTS

**Figure 1:**
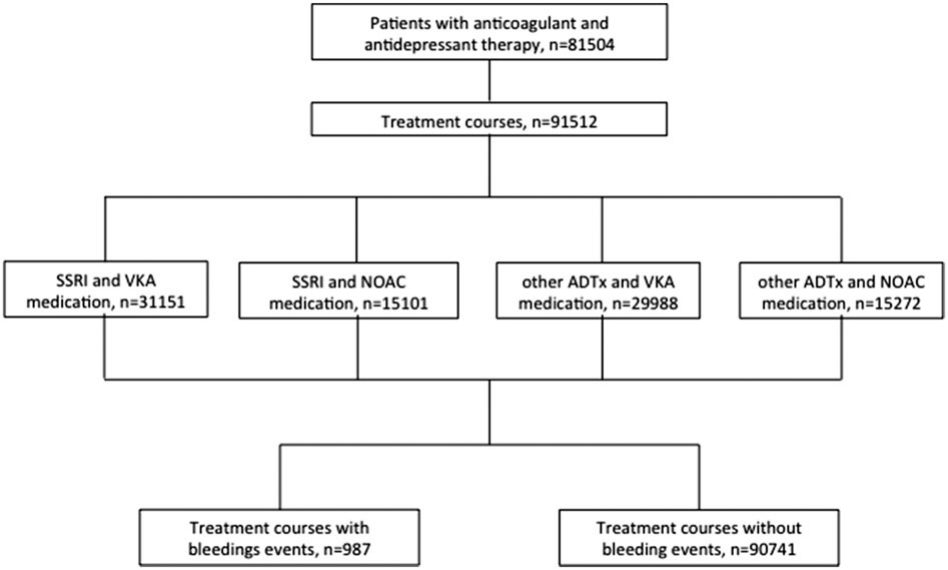
Number of patients, treatment courses, and clinical events with oral anticoagulants and selective serotonin receptor inhibitor (SSRI) or other antidepressant medicine (ADTx)

**Figure 2:**
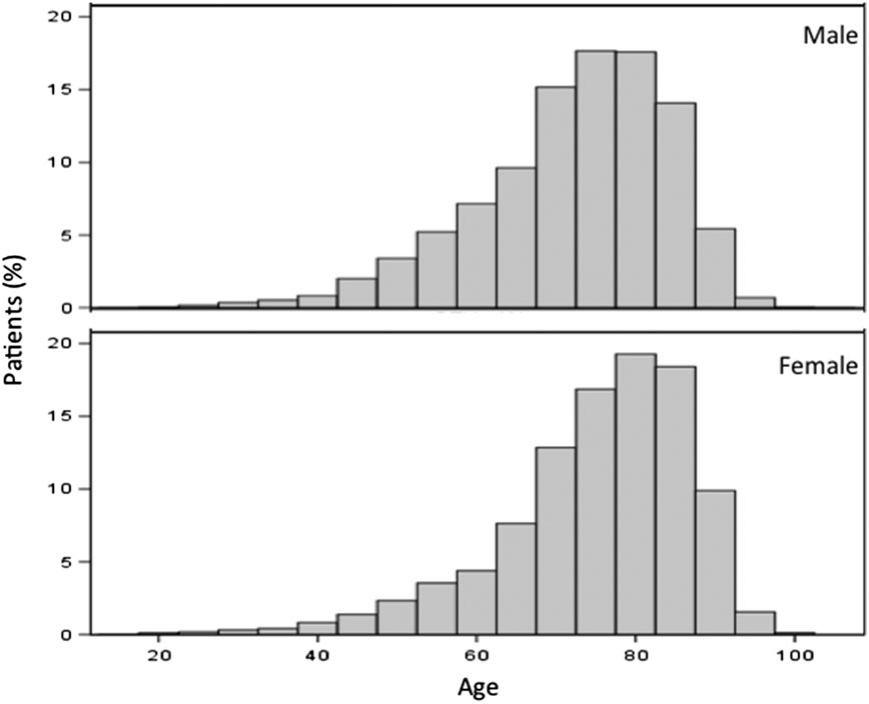
Age distribution of patients

Data from 50 196 female and 31 308 male patients with a median age of 76 years (interquartile range 68‐83 years) were analysed (Figure 1). Figure 2 presents the age distribution of patients under treatment; 7560 patients were without other concomitant medication; 18 427 patients had a co‐medication for diabetes, 71 537 for a CV indication, and 25 770 received a treatment for PD.

In total, 91 512 patient‐treatment courses with a maximum of one switch between anticoagulant and antidepressant therapy were analysed; 987 hospitalisations with bleeding events in discharge diagnoses were detected from 892 patients. Up to four relevant hospitalisations per patient were observed. The most frequent bleeding event was GI bleeding with 588 cases (59.6%), followed by cerebral haemorrhage with 344 (34.8%), and bleeding anaemia with 55 events (5.6%). (Table 1).

**Table 1:** Anticoagulant and antidepressant treatment combination and events per patient year

		Patient Years	Gastrointestinal Bleeding (n)	Gastrointestinal Bleeding (e/py)	Cerebral Haemorrhage (n)	Cerebral Haemorrhage (e/py)	Bleeding Anaemia (n)	Bleeding Anaemia (e/py)
SSRI	VKA	27221	193	0.0071	158	0.0058	21	0.0008
	NOAC	10179	118	0.0116	47	0.0046	11	0.0011
Other ADTx	VKA	20230	175	0.0087	104	0.0051	20	0.0010
	NOAC	8251	102	0.0124	35	0.0042	3	0.0004

Abbreviations: ADTx, antidepressant medicine; e/py, events per patient year; NOAC, non‐vitamin K antagonist; py, patient years; SSRI, selective serotonin reuptake inhibitor; VKA, vitamin K antagonist.

### 3.1 Bleeding events with NOAC/VKA and SSRI/other ADTx

The risk of bleeding events was similar between SSRI and other ADTx when combined with oral anticoagulants (*p* = 0.51). The concomitant treatment of patients with an antidepressant (SSRI or ADTx) and NOAC was associated with an increased risk for a bleeding event compared with cotreatment of an antidepressant with VKA with a RR of 1.21 (95% CI: 1.05‐1.40; *p* = 0.0097).

The risk for GI bleeding per patient per year was significantly higher in patients with NOAC compared with those with VKA with a RR of 1.53 (95% CI: 1.28‐1.84; *p* < 0.0001).

Cerebral haemorrhage was observed more often in patients with VKA compared with those with NOAC; however, this difference was not statistically significant (*p* = 0.12).

Patients with SSRI and VKA medication had a twofold higher risk of bleeding anaemia compared to patients with other ADTx and NOAC (0.0008 vs 0.0004 event risk per patient year). The interaction between antidepressant and anticoagulant medication for bleeding anaemia was augmented when patients were treated with SSRI and NOAC or other ADTx and VKA (0.0011 and 0.0010 event risk per patient year, respectively; *p* = 0.0465).

## 4 DISCUSSION

This retrospective population‐based cohort study has investigated the clinical outcome of concomitant anticoagulant medicine with antidepressant therapy prescription during a maximum observation period of 5 years and presents two major findings.

Our first finding is that patients with SSRI had a similar risk for bleeding events as patients with other antidepressant therapy receiving NOAC or VKA. Second, bleeding events in patients with SSRI or other antidepressant therapy who received a coprescription with NOAC were higher than those recorded for co‐medication with VKA. The risk of bleeding events in patients with concomitant treatment of SSRI and NOAC has not been reported yet. An increased bleeding risk has been described for patients treated with SSRI and VKA compared with patients receiving other antidepressants in previous case reports and studies.^8^, ^9^, ^10^, ^11^ This has been reported for upper GI bleedings as well as for the risk of cerebral haemorrhage.^4^, ^11^, ^12^

A drug–drug interaction may cause an increased bleeding risk, when anticoagulant and antidepressant medications are combined. A pharmacokinetic interaction between SSRI with VKA through the competitive inhibition of cytochrome enzymes (CYP2C9) has been described.^13^ Likewise, a potential mechanism for increased bleeding with SSRI may be explained by an additive pharmacodynamic effect on the inhibition of platelet aggregation.^14^, ^15^, ^16^, ^17^ In contrast to the assumption that SSRI could result in increased bleeding in patients with NOAC, we could not detect a higher number of selected bleeding events in the group of patients receiving SSRI in this real world analysis compared to other ADTx. Importantly, the increase in bleeding events in patients with NOAC compared with VKA was independent from the class of drugs and occurred also with other antidepressant medicines. This statistical difference was mainly driven by the risk for GI bleedings.

Our results are in line with a recent register study from Sweden that showed a higher risk of GI‐bleeding events in patients treated with NOAC compared with those receiving VKA.^18^ The Dresden NOAC registry also reported that patients receiving NOAC had a greater GI‐bleeding risk, in particular in the lower GI tract, when compared with VKA recipients.^19^ In contrast, a previous real‐world study from Texas showed a smaller number of GI‐bleeding events in patients taking NOAC than those receiving VKA; however, the number of patients treated with NOAC was small.^20^ Consistent with our data, recent studies have demonstrated that cerebral haemorrhage occurred less often in patients on NOAC treatment compared with VKA.^18^, ^21^

Conflicting results have been reported concerning the bleeding risk of patients receiving SSRI treatment in previous observational retrospective studies, which might be explained by different applied methodologies, health care system, selection bias, or follow‐up periods. In the general population, an increased risk of GI bleeding or blood transfusion was associated with SSRI intake.^22^, ^23^, ^24^, ^25^, ^26^, ^27^, ^28^ However, recent studies showed that a prestroke SSRI exposure was not associated with increased cerebral haemorrhage.^29^ In addition, investigations in patients undergoing cardiac surgery showed that SSRI exposure was not associated with increased bleeding risk or blood transfusion.^30^, ^31^, ^32^, ^33^ Our findings are also at variance with results from previous studies that have reported an increased bleeding risk in patients on SSRI treatment.^34^, ^35^, ^36^ Large controlled clinical trials may be necessary to address this safety concern accordingly.

Co‐medication of SSRI with NOAC or VKA had no impact on bleeding events in hospital discharge diagnoses compared with coprescription of other antidepressant medicines in our cohort. This study has not distinguished between the different medicines among the class of NOAC as the number of events was relatively small and any subgroup analysis subject to bias. Further, we cannot rule out that minor bleeds are affected by concomitant SSRI or other antidepressant therapy prescription. Such events are not recorded in our database. However, our analysis argues that concomitant prescription of SSRI with anticoagulants confers a similar safety regarding the occurrence of major and clinically relevant bleeds as a coprescription of other antidepressants. Based on robust hospital discharge diagnoses, we conclude that prescription of SSRI with anticoagulants in clinical practice does not require dose adjustment to avoid major bleeding events.

Our study has several limitations. ICD‐10 coding of hospital discharge diagnosis was used to identify bleeding events in the database and the clinical diagnosis or case severity was not adjudicated for. In addition, the analysis of bleeding events is limited to patients who have been discharged following hospitalisation and does not include events from ambulatory care. An analysis of causes of death is also not available. Confounding may arise from limited information on comorbidities from disease‐specific drugs using ATC codes. Information of prescribed medication is also limited to those medicines, which are reimbursed by the health care providers. Also, in the present study, no information about concomitant nonsteroidal anti‐inflammatory drug intake was available, which has been shown to have an impact on bleeding events when combined with SSRI or anticoagulants.^37^, ^38^ Furthermore, we were not able to assess adherence to the medications for which prescriptions were filled.

## 5 CONCLUSION

Co‐medication of SSRI with NOAC or VKA has no substantial impact on major and clinically relevant bleeding events compared with cotreatment of these oral anticoagulant agents with other antidepressant medicines as assessed from retrospective review of hospital discharge diagnoses. This argues against a clinically relevant increase in the risk for major bleedings associated with SSRI in patients receiving continuous oral anticoagulation.

## CONFLICT OF INTEREST

None declared.

## AUTHOR CONTRIBUTIONS

S. Sheikh Rezaei planned and conducted the study and wrote the paper with input from all authors. M. Mittlböck prepared the data and performed the statistical tests. B. Reichardt collected data and prepared the data. M. Wolzt planned and conducted the study and supervised the project. All authors discussed the results and contributed to the final manuscript. All authors report no financial support or relationships that could be construed as a conflict of interest.

## AVAILABILITY OF DATA AND MATERIALS

Derived data supporting the findings of this study are available from Data Clearing House of Medical University of Vienna on request.
